# Performance of an eHealth (NOMHAD) System Comprising Telemonitoring, Telenotification, and Telecoaching for Patients With Multimorbidity: Proof-of-Concept Study

**DOI:** 10.2196/32205

**Published:** 2022-03-11

**Authors:** Mathieu Chacornac, Aurélien Faoro, Joëlle Texereau, Catherine Billoet, Stéphane Hominal

**Affiliations:** 1 Department of Cardiology Centre Hospitalier Annecy-Genevois Pringy France; 2 Department of Respiratory Medicine Cochin Hospital, Assistance Publique-Hôpitaux de Paris Université de Paris Paris France; 3 VitalAire Air Liquide Health Care Gentilly France; 4 Air Liquide Santé International Paris France; 5 Department of Pneumology Centre Hospitalier Annecy-Genevois Pringy France

**Keywords:** multimorbidity, telehealth, telemonitoring, chronic disease, chronic heart failure, chronic obstructive pulmonary disease, diabetes

## Abstract

**Background:**

Management of patients with multiple chronic diseases is a growing public health challenge, especially in rural sectors where access to physicians may be limited. Connected medical devices monitoring vital signs, associated with eHealth program and structured telephone support, may improve complex patient management through early detection of disease complications, positive impact on patients’ health, and health resources consumption optimization.

**Objective:**

The aim of this study was to evaluate the technical performance and user experience of the NOMHAD eHealth system in patients with multimorbidity.

**Methods:**

This was a pilot, single-arm, interventional study. Patients with multimorbidity with any combination of chronic heart failure (CHF), chronic obstructive pulmonary disease, and diabetes were followed for 80-100 days using the NOMHAD eHealth system. This system used connected devices telemonitoring symptoms and vital signs (eg, body weight, oxygen saturation, pulse rate, blood pressure, and blood glucose), associated with structured telecoaching and educational support by call center nurses. An overall risk indicator (ORI) was automatically computed after each data teletransmission. The ORI was color coded; green indicated no action required; yellow, orange, and red (low to high priority, respectively) generated telenotifications and indicated to the nurses the need for a telecoaching action. Each ORI was calculated by combining 7 clinical stability system indicators based on symptom questionnaires and vital signs. Technical accuracy of the system was assessed by comparing system-generated ORIs with ORIs recalculated from raw data. Ease of use, usefulness, satisfaction, and acceptability of the system were assessed through patient adherence to self-assessments, and through self-administered questionnaires to patients, call center nurses, and physicians.

**Results:**

A total of 23 patients were enrolled in this study and participated between April 2016 and March 2017 at 5 study centers in France. All patients were successfully equipped and evaluable for analysis. Mean age was 68.5 (SD 10.4) years and most patients were men (n=20). The most common multimorbidity was CHF + diabetes (n=15), followed by patients with all 3 diseases (n=5). Mean effective follow-up was 78.7 (SD 24.2) days. The system generated 6263 ORIs, as several ORIs could be generated on a single day for any patient. Overall system sensitivity was 99.2% (95% CI 98.9-99.4) and overall specificity was 91.3% (95% CI 87.7-94.1). Most patients (20/23, 87%) were satisfied with the system and agreed that it helped them to better understand and manage their diseases, and 19/23 (83%) valued the nurse regular contacts. Nurses and physicians were generally satisfied with the system and considered it useful. All users indicated they would agree to long-term use of the system.

**Conclusions:**

This study provides evidence that the NOMHAD eHealth system is accurate, acceptable, informative, and feasible for patients with multimorbidity, supporting further investigation of its clinical benefits.

**Trial Registration:**

Agence Nationale de Sécurité du Médicament et des Produits de Santé 2015-A01106-43; https://ictaxercb.ansm.sante.fr/Public/index.php

## Introduction

### Background

The management of patients with chronic diseases is a major public health challenge that continues to grow as the numbers of these patients continue to increase [1]. In developed countries, advances in medical care and consequently longer life expectancies have contributed to a steady rise in the numbers of patients with chronic diseases such as chronic heart failure (CHF), chronic obstructive pulmonary disease (COPD), and diabetes, all of which are among the leading causes of death [2]. These patients now account for a major share of many national health care budgets. In France, it was estimated that approximately 80% of the national health care budget in 2012 was dedicated to care for patients with chronic illnesses [3].

Medical deserts due to uneven geographic distribution of health care practitioners are a reality for many patients in France. Despite a steady increase in the number of physicians, many will retire in the next few years, which will further decrease health care availability in some territories. This trend is exacerbated by a large disparity across France in the numbers of physicians available per person, which is highest near Paris [4].

Faced with the rising prevalence and burden of chronic illnesses, health care systems have recognized that traditional reactive medical care involving repeated hospitalizations for disease complications is unsustainable [5,6]. Thus, alternative remote methods of managing patients with chronic diseases are needed to promote and stabilize patient health at home, while minimizing the risk of hospitalization and the burden on health care resources. Chronic disease telemanagement programs aim to foster continuity between hospital and home care, prevent disease worsening and the onset of complications, prevent or reduce hospitalizations, ensure appropriate and timely treatment when needed, educate patients to empower disease self‑management, and optimize physician involvement. The ultimate goal for patients is to live safely at home while continuing to receive optimal care.

French national recommendations have called for the development of such telemedicine interventions in patients with CHF, COPD, and diabetes—the 3 chronic diseases that impose the highest burdens on the health care system. The sudden episodes of deterioration that characterize these diseases, such as cardiac decompensations in CHF, acute exacerbations in COPD, and major hypoglycemic events in diabetes, require hospitalization and very often a second hospitalization soon after discharge. Some of these hospitalizations and rehospitalizations may be partially preventable if symptom worsening is detected and managed early [7,8].

Home telemonitoring programs are among the solutions currently investigated for remote disease management. The intent is to provide high-quality health care to patients while they live at home. In patients with CHF, remote telemonitoring programs have been shown to reduce mortality [9-13], hospitalizations [9,10,12-14], emergency care unit visits [9], and health care costs [9]. In a review of transitional care interventions, programs that included home visits and multidisciplinary interventions reduced mortality and all-cause readmissions, but telemonitoring had no beneficial effect [15]. In patients with COPD, the benefits of telemonitoring programs versus usual care have been less clear. In the recent COPD Patient Management European Trial (COMET) that investigated a COPD disease management intervention that included remote telemonitoring in patients with severe COPD, acute care hospitalization days and mortality were significantly lower in the disease management group [16]. Other analyses have found inconsistent impact of telemonitoring on hospitalizations [17-19] and quality of life [17-20], a trend toward reduced costs [20,21], improved physical activity but no effect on physical capacity or dyspnea [22], and no effect on length of hospital stay or mortality [19]. Most reviewers concluded that despite some encouraging results, additional evidence from larger, high-quality studies is needed before definitive conclusions and recommendations can be made on the benefits of telemonitoring programs for patients with COPD [12,19,20,22,23]. For patients with diabetes, clinical trials and systematic reviews have consistently demonstrated that telemonitoring programs are feasible and can sustain improved glycemic control over time versus usual care [24-26]. Evidence for other benefits, such as reduced BMI [24], reduced use of health care resources and costs [26,27], and lower mortality and cognitive decline in older patients, has been modest or inconsistent [27].

Some of the difficulty in interpreting the results of the many studies investigating the benefits of telemonitoring programs in patients with chronic diseases stems from the heterogeneity in the programs and in the patient populations studied. Most telehealth programs have focused on a single chronic disease, but the reality is that many patients with chronic diseases have more than 1 disease [28-30]. The overall prevalence of such multimorbidity was 23% among all patients in a national health care database in Scotland [28], whereas the prevalence of multimorbidity is much higher (>60%) in the growing European population of patients over the age of 65, who tend to accumulate chronic conditions as they age [28,30]. Multimorbidity is well described in patients with CHF, COPD, or diabetes. In another study conducted in Scotland, 42% of patients with CHF also had diabetes and 26% also had COPD [27]. Similarly, 39% of patients with COPD had CHF and 22% had diabetes, and 45% of patients with diabetes had CHF and 15% had COPD [27]. Thus, telehealth and telemonitoring programs designed for use in patients with a single chronic disease may be limited in their capacity to provide benefits to patients with multiple chronic conditions or comorbidities.

### The NOMHAD eHealth System: Overview

The NOMHAD eHealth system, consisting of a web interface for physicians and nurses (NOMHAD Chronic) and an app for patients (NOMHAD Mobile), is derived from a Spanish system and is specifically designed to handle patients with multiple chronic diseases [31]. This system aims to provide telemonitoring by means of detecting health status deterioration based on connected devices and telecoaching based on educational support by dedicated call center nurses.

### Study Objectives

In this exploratory study, the technical performance of the NOMHAD eHealth system was evaluated in patients with multimorbidity with any combination of CHF, COPD, and diabetes. The ease of use, usefulness, satisfaction, and acceptability of the system were also investigated from the perspectives of the patients, call center nurses, and physicians.

## Methods

### Study Design and Objectives

This was a pilot, multicenter, nonrandomized, single-arm, open-label, uncontrolled interventional study to evaluate the performance of the NOMHAD eHealth system of telemonitoring, telenotification, and telecoaching (Agence Nationale de Sécurité du Médicament et des Produits de Santé [ANSM] registration number [ID-RCB]: 2015‑A01106-43). The system was evaluated over 3 months of follow-up in a single group of approximately 30 ambulatory patients with multimorbidity.

The primary objective of the study was to evaluate the technical performance of the NOMHAD eHealth system in this population. This objective was to be achieved by comparing patient overall risk indicators (ORIs) generated by the system with those recalculated on the basis of raw data teletransmitted to the software platform. Secondary objectives were to evaluate the impressions and perspectives of NOMHAD eHealth system users (patients, study physicians, and nurses) using different self‑questionnaires about overall ease of use, usefulness, satisfaction, and acceptability. Feasibility of the intervention, technological performance of the system, clinical events, adverse events (AEs), and adverse device events were also assessed.

### Ethics Approval

The study protocol (2015-A01106-43) and subsequent amendments were approved by a local independent ethics committee (Comité de Protection des Personnes Lyon Sud-Est IV) and the French Medicine Agency (ANSM). The study was conducted in compliance with Good Clinical Practice guidelines, the Declaration of Helsinki, the EU Council Directive 93/42/EEC regarding Medical Devices, and International Standard ISO 14155. Study protocol, benefits, and risks were explained to participating patients, who were required to provide written informed consent.

### Study Participants

Patients were recruited and enrolled at 5 study centers in France (Rhône-Alpes region). Adults over the age of 18 years with at least two chronic diseases among CHF, COPD, and diabetes and with at least one hospitalization for acute decompensation of CHF or exacerbation of COPD during the year prior to inclusion were eligible to participate in the study. Patients were also required to speak and understand French and have a landline phone. To be enrolled, patients had to meet the French Health Authorities (Haute Autorité de Santé) diagnosis criteria [32], briefly summarized in Table 1. No biological or clinical examinations were required at study entry, but the results of the most recent examinations performed per routine practice were to be reported. Patients were excluded if they were pregnant or breastfeeding, were institutionalized, had a life expectancy of less than 3 months, had received within the past 6 months or were receiving chemotherapy or radiotherapy for cancer, required dialysis for chronic renal insufficiency, had a condition likely to interfere with study procedures, were not covered by the French National Health System, or had participated within the past 30 days or were participating in another interventional trial.

**Table 1:** Main diagnosis criteria^a^ for enrollment of patients with chronic obstructive pulmonary disease, diabetes, or chronic heart failure.

Disease	Criteria
Chronic obstructive pulmonary disease	Clinical signs AND exposure to a known risk factor AND forced expiratory volume in 1 second/forced vital capacity ratio <70% after bronchodilator
Diabetes	Blood sugar >1.26 g/L (7.0 mmol/L) after 8-hour fasting (assessed twice) OR diabetes symptoms with blood sugar >2 g/L (11.1 mmol/L) OR blood sugar >2 g/L, 2 hours after a 75-g oral glucose challenge
Chronic heart failure	Clinical symptoms or signs AND objective evidence of structural or functional cardiac abnormality at rest. Systolic heart failure OR heart failure with preserved ejection fraction

^a^Criteria from the French National Health Authorities (Haute Autorité de Santé [32]).

### NOMHAD eHealth System

**Figure 1:**
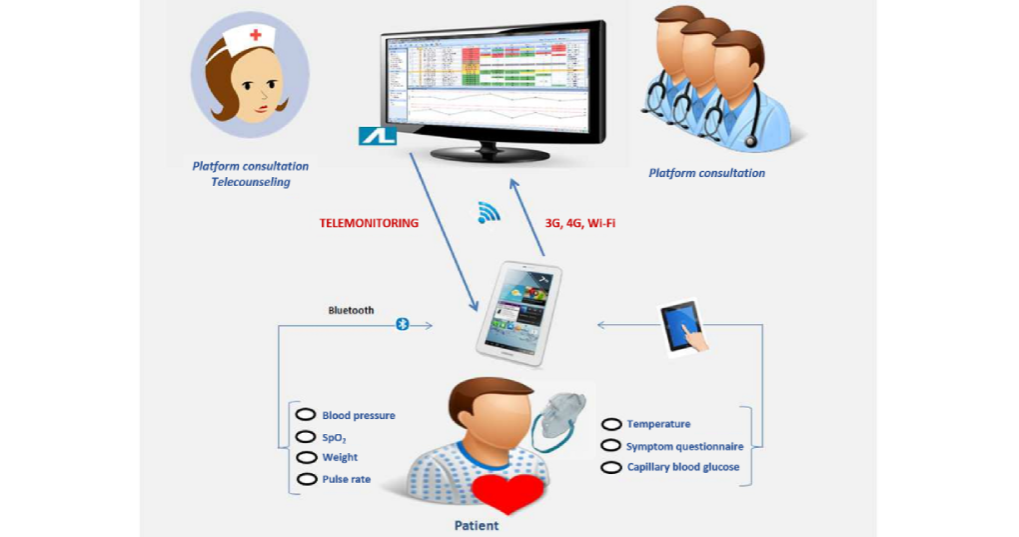
Overview of the NOMHAD eHealth system. The NOMHAD eHealth system consists of the NOMHAD Chronic software platform and the remote NOMHAD Mobile app installed on an interactive tablet, which is used by the patient to enter vital sign data, either manually or through connected devices, and answer questionnaires. These data are transferred to the NOMHAD Chronic software platform using available 3G, 4G, or Wi-Fi connections. The NOMHAD eHealth system is a global system including health care technicians, call center nurses, study physicians, and referring physicians. 3G: third generation; 4G: fourth generation; SpO_2_: arterial oxygen saturation; Wi-Fi: wireless fidelity.

The NOMHAD eHealth system employs the NOMHAD Chronic software platform (version 1.6.4; Connected Health Services) and the remote NOMHAD Mobile app (version 1.2.1) installed on an interactive tablet (Figure 1). Through NOMHAD Mobile, patients self-measure their vital signs through connected devices (or manually), provide additional health status–related information by answering questionnaires, and transmit the information to the central database.

The NOMHAD Chronic software platform consists of a web interface for physicians to define personalized patient care plans and browse patient data, and a call center station web interface for nurses (skilled home health care provider nurses working at the VitalAire France nurse call center) to browse patient data, prioritize nurse activities, and report telecoaching actions.

NOMHAD Chronic monitors up to 7 clinical stability systems indicators that inform about chronic disease/health status parameters according to patient medical conditions and individual-specific thresholds: hemodynamic stability, respiratory stability, pain/sleep quality, diet/diuresis, tissue integrity, cognitive system/mood, and medication.

The software platform incorporates an algorithm that calculates the ORI to guide the nurse in prioritizing telecoaching actions. ORIs are color coded, enabling immediate identification of the patients to be contacted and their level of priority. A green ORI indicates that no action is required, whereas yellow (low priority), orange (medium priority), and red (high priority) ORIs generate telenotifications and indicate to the call center nurse the need for a telecoaching action, from simple reinforcement, repeating measurements and adapting the care plan, to advising the patient to call an emergency medical service. The ORI is calculated by combining the 7 clinical stability system indicators, which are themselves calculated by combining the results of symptom questionnaires and vital signs. An individual ORI is calculated by the algorithm every time a new value of any monitored parameter is transmitted by the patient. Therefore, several ORIs and corresponding telenotifications may be generated on a single day for a given patient.

### Patient Interventions

**Figure 2:**
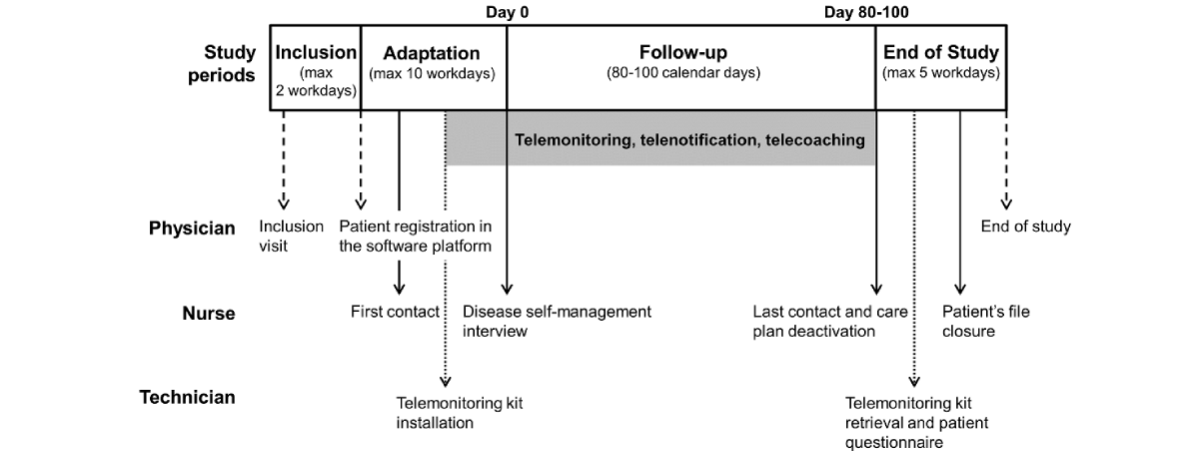
Study flow for the patient. The total study duration for a patient was approximately 15 weeks.

Patients participated in the study over a period of approximately 3 months (Figure 2). Inclusion was followed by an adaptation period during which the telemonitoring kit was installed by a home health care provider technician and first used autonomously by the patients in their home. The follow-up period began with a phone interview with the nurse to evaluate the patient’s self-management skills specific for their chronic diseases, establish objectives for improvement, and collect patient lifestyle data. During the follow-up period, patients performed regular self-assessments of their health status according to their chronic diseases. Patients used the tablet to enter their answers to health questionnaires and the devices connected to the tablet to measure vital signs (eg, blood pressure, pulse rate, arterial oxygen saturation, daily blood glucose, body weight depending on their medical condition, and personalized care plan) and transmit them to the NOMHAD Chronic system. The standard patient care plan (consisting of self‑measurements twice a week and questionnaires once a week in stable state) could be personalized according to patient needs, the system notifications generated, and study physician assessment.

Patients were contacted by a nurse at regular intervals (at least every 15 days for the first 2 months) and when the system generated a telenotification. Patients could also contact a nurse whenever necessary during regular business hours via a toll-free phone number, for instance, to report a hospitalization. Through the NOMHAD Mobile app, patients could consult disease self‑management support modules installed on their tablet and could voluntarily participate with other study patients in online video classroom sessions conducted by the nurses. The objective of these sessions was to provide patients with supplementary information about their chronic diseases and their management while taking advantage of group dynamics. The telemonitoring program could be suspended temporarily by a call center nurse for patient’s personal reasons or during a hospitalization.

Nurses from the call center were trained to provide support by phone to patients with chronic diseases and were connected to the NOMHAD Chronic system to manage telenotifications (according to their priority level) during regular working hours. They performed standardized telecoaching actions, such as advising patients to seek physician consultation or emergency care, according to the type and severity of the telenotifications.

Physicians could access patient data at any time; were prompted to log-on to the system during patient consultations; and could modify patient medical data, medications, procedures, system threshold values for health status parameters, and recorded any AE. After a follow-up period of 80-100 days, the care plan was deactivated, equipment was retrieved, and study physicians completed the electronic case report form.

### Outcomes and Evaluation Methods

For the primary performance criterion, the sensitivity and specificity of the ORIs generated during the follow-up period were assessed by comparing the ORIs generated by the NOMHAD system using a specific algorithm (ORI_NOMHAD_) with those expected when recalculated from the raw data teletransmitted to the software platform (ORI_EXP_) based on its algorithm specifications. Each combination of ORIs (ORI_NOMHAD_ vs ORI_EXP_) obtained was categorized as follows: if the ORI_NOMHAD_ and ORI_EXP_ were both green, the scoring was considered “correctly not telenotified” (ie, true negative [TN]); if the ORI_NOMHAD_ and ORI_EXP_ colors were the same for yellow, orange, or red, the scoring was considered “correctly telenotified” (ie, true positive [TP]); if the ORI_NOMHAD_ priority color was lower than the ORI_EXP_ color, the scoring was considered “incorrectly identified as lower priority” (ie, false negative [FN]); and if the ORI_NOMHAD_ priority color was higher than the ORI_EXP_ color, the scoring was considered “incorrectly identified as higher priority” (ie, false positive [FP]). Secondary performance criteria were the numbers and frequencies of telenotifications and the sensitivity (defined as TP/[TP + FN] × 100) and specificity (defined as TN/[TN + FP] × 100) of the clinical stability system indicators. The overall sensitivity and specificity were calculated from all ORI recordings.

At the end of the study, patients, nurses, and physicians completed user-specific self‑questionnaires to assess their perspectives on the ease of use, usefulness, and satisfaction of the system. These self-questionnaires were developed by the sponsor specifically for the study. System acceptability was assessed from the actual duration of follow-up and the duration of effective follow-up (after deduction of the temporary suspensions periods). System feasibility was assessed from the numbers of patients refusing or discontinuing the program for any nonmedical reasons. Safety was evaluated from device deficiencies and AEs starting during the study follow-up period.

### Sample Size Estimation

An appropriate sample size could not be calculated because no assumptions could be made prior to the start of the trial regarding the primary technical performance criterion in patients with multimorbidity. It was estimated that approximately 30 evaluable patients would be sufficient to establish preliminary descriptive conclusions for the primary criterion.

### Statistical Methods

Statistical analyses were performed using SAS software, version 9.2 (SAS Institute). Summary statistics for continuous variables were number of observations, mean, SD, median, first and third quartiles (Q1 and Q3), minimum and maximum values, and the number of missing information. Summary statistics for dichotomous or categorical variables were the number and percentage of each of the scores or categories with 95% CIs calculated using the exact binomial distribution for percentages, where applicable. The agreement between all pairs of ORIs generated by the software platform and by those recalculated on the basis of the raw data teletransmitted (each time the algorithm was run by the software platform) was evaluated with SAS by calculating the weighted Fleiss–Cohen κ coefficient for all pairs of ORI scores and by testing the null hypothesis that this coefficient was equal to 0 (ie, no agreement apart from pure chance agreement existed between both scorings). Results are presented for all patients who were enrolled in the study.

### Amendments to the Study Protocol

After the study began, the study protocol was amended to stop recruitment before the planned number of enrolled patients was reached. This was done because the recruitment period had already been extended and it revealed that investigators had reached their maximal recruitment potential based on the study inclusion criteria.

## Results

### Study Patients

A total of 23 patients were enrolled in the study and participated between April 2016 and March 2017 at 5 study centers. Three patients did not complete the study: 1 withdrew consent, 1 moved into a care facility that precluded study procedures, and 1 requested to discontinue telemonitoring. Thus, 20 patients completed study over a mean duration of 99.7 (SD 18.9) days. There were 18 patients with at least one minor protocol deviation, most were for missing data (n=15) or time window deviations (n=8). No major protocol deviations (defined as those affecting the minimal 2-month required self-recordings) occurred, except the 7-day follow-up of the patient who withdrew consent.

The mean age of the patients in the study was 68.5 (SD 10.4) years and most patients (20/23, 87%) were men (Table 2). The mean BMI was 33.9 (SD 7.2) kg/m^2^ with a range of 24.5-54.6 kg/m^2^. Most patients did not live alone, had a caregiver, and declared having an occupational activity outside the home and regular physical activity.

**Table 2:** Patient demographics, lifestyle, therapy, and vital signs at study entry.

Characteristic		All patients (N=23)
Sex, male, n (%)		20 (87)
Age (years), mean (SD)		68.5 (10.4)
BMI (kg/m^2^), mean (SD)		33.9 (7.2)
Never or former smoker, n (%)		21 (95)^a^
Living alone^b^, n (%)		8 (35)
Presence of a caregiver^b^, n (%)		13 (57)
Occupational activity outside the house^b^, n (%)		14 (61)
Regular physical activity^b^, n (%)		15 (65)
Difficulties in understanding the diet^b^, n (%)		11 (48)
Regular body weight monitoring^b^, n (%)		17 (74)
Blood glucose self-monitoring (patients with diabetes)^b^, n (%)		18 (86)^c^
**Therapy^d^, n (%)**		
	Insulin	11 (48)
	Home oxygen therapy	3 (13)
	Home noninvasive ventilation	5 (22)
Systolic blood pressure (mmHg), mean (SD)		125.4 (23.3)
Diastolic blood pressure (mmHg), mean (SD)		72.8 (13.6)
Pulse rate (beats/minute), mean (SD)		79.7 (19.0)

^a^N=22.

^b^As declared by the patient to the call center nurse.

^c^N=21.

^d^As recorded in the NOMHAD eHealth system. Multiple responses were allowed. % = (n row/n nonmissing) × 100, except for multiple responses where % = (n row/N group) × 100.

The most frequent multimorbidity was CHF + diabetes (n=15; Table 3). Five patients had all 3 diseases (CHF + diabetes + COPD). The severity of CHF was most frequently reported as New York Heart Association class II (11/19, 58%) or III (7/19, 37%), and patients with CHF (n=21) had a mean left ventricular ejection fraction of 44.5% (SD 13.5%). Patients with COPD (n=6) had a mean forced expiratory volume in 1 second of 55.0% (SD 16.9%) predicted (n=6). Patients with diabetes had mainly type 2 diabetes (n=19), with a mean last measured glycosylated hemoglobin (HbA_1c_) of 7.7% (SD 1.6%) (n=18). Micro- and macro-angiopathic complications were reported in 11 and 4 patients, respectively. Overall, patients had a mean of 6.5 (SD 2.9) concomitant diseases that were recorded by their study physician at inclusion in addition to their targeted chronic diseases.

**Table 3:** Patient multimorbidity.

Multimorbidity profile of study patients		All patients (N=23)
**Patient’s main chronic disease profile, n (%)**		
	CHF^a^ + COPD^b^	2 (9)
	CHF + diabetes	15 (65)
	COPD + diabetes	1 (4)
	CHF + COPD + diabetes	5 (22)
**Number of concomitant diseases at inclusion, mean (SD)**		6.5 (2.9)
Comorbidities^c^, n (%)		
	Dyslipidemia	16 (70)
	Arterial hypertension	16 (70)
	Chronic respiratory failure	7 (30)
	No comorbidity ticked	3 (13)

^a^CHF: chronic heart failure.

^b^COPD: chronic obstructive pulmonary disease.

^c^As recorded in the NOMHAD eHealth system. Multiple responses were allowed. % = (n row/n nonmissing) × 100, except for multiple responses where % = (n row/N group) × 100.

### System Performance

For all patients, the NOMHAD eHealth system generated a total of 6263 ORIs that could be recalculated from the raw data teletransmitted during the 3-month follow-up period. Among these ORIs, 294 (4.69%) were green, 4480 (71.53%) were yellow, 1441 (23.01%) were orange, and 48 (0.77%) were red. ORI proportions by color were similar for each chronic disease individually.

For all patients, there was good agreement between the telenotifications generated by the NOMHAD eHealth system and those recalculated from the raw data. In the primary analysis, overall sensitivity was 99.2% (95% CI 98.9-99.4) and overall specificity was 91.3% (95% CI 87.7-94.1). Sensitivity and specificity results were similar for each chronic disease individually. Agreement evaluation of all ORI pairings yielded a weighted Fleiss–Cohen κ coefficient of 0.975 (95% CI 0.969-0.981; *P*<.001), indicating good agreement between all pairs that was not due to pure chance. Kappa coefficients for each chronic disease individually were similarly high (≥0.975).

### System Effectiveness

During the 3-month follow-up period, all patients generated at least one telenotification and triggered, therefore, nurse telecoaching actions. The highest proportions of telenotifications were yellow and orange (ie, low and moderate priority, respectively). All 23 patients generated at least one yellow ORI-low priority telenotification (with a mean of 190 per patient over the 3-month period), 17 patients generated at least one orange ORI-moderate priority telenotification (with a mean of 83 per patient), and 7 patients generated at least one red ORI-high priority telenotification (with a mean of 6 per patient).

The management of the telenotifications resulted in a median of 21 nurse contacts per patient (Q1-Q3, 6-49). The call duration lasted 11-20 minutes at least once for most patients (22/23, 96%). The mean time interval between a telenotification during business hours and registration of telecoaching action was 68.3 (SD 63.6) minutes.

Physicians did not change the predefined parameters or thresholds. On average, physicians connected 3 times per patient for approximately 30 minutes in total, during the 3-month follow-up.

### End User Perspectives

#### Patients

Most (21/23) patients felt that health care technicians spent sufficient time installing the study devices and training them to use the eHealth system. Most patients were satisfied with the coaching they received from the nurses.

Eighteen patients agreed or strongly agreed that the information contained in the app was useful in helping them better understand their illnesses and 14 patients agreed or strongly agreed that it helped them better manage their symptoms. Patient contact with the call center nurses was considered helpful, as 16 patients agreed or strongly agreed that it helped them become more independent in managing their illnesses, and 17 patients agreed or strongly agreed that they received effective advice when needed. Sixteen patients participated in at least one online video classroom session. However, only 7 patients agreed or strongly agreed that the conferences helped them to better cope with their illnesses through sharing their experience with other patients.

Overall, 20 patients agreed or strongly agreed that they were generally satisfied with the eHealth system, 10 patients agreed or strongly agreed that it improved their relationship with their study physician, and 15 patients agreed or strongly agreed that they would be prepared to use it long term.

#### Nurses

Nurses (n=3) strongly agreed that their involvement in the study helped them to communicate more effectively with patients and agreed that it helped patients to better understand how to manage their diseases with self-measurement and self-management tools. All nurses strongly agreed that they would be prepared to use the system long term. Overall, they ranked the remote vital signs measurements and the planned regular phone calls with the patients to be the best components.

#### Study Physicians

Study physicians (n=5) agreed that the software platform was somewhat or fairly intuitive and user-friendly, and 4 agreed that its content met their expectations. One of them modified the standard monitoring patient care plan of 1 or more patients and felt this was a medically relevant action and easy to perform. Three physicians agreed that the telemonitoring and telecoaching interventions by the nurses resulted in physicians seeing only those patients with a genuine need, whereas all agreed that the frequency of notifications and patient consultations was sufficient.

Three physicians agreed or strongly agreed that the information available on the software platform enabled them to better understand their patients’ disorders and to better manage their patients. Four of the physicians agreed or strongly agreed that they could better anticipate complications affecting patients with multiple morbidities and that their patients were better equipped with self-measurement and self-management tools. Four agreed or strongly agreed that it improved their relationship with their patients and 4 were fairly or very prepared to use the system long term. The study physicians ranked the vital signs, the availability of the telemonitoring platform, and the telecoaching actions among the more preferred components.

### System Acceptability, Feasibility, and Technical Performance

Acceptability, as assessed by the extent to which planned events were completed, was generally high (Table 4). An average of 87.3% (SD 34.6) of the total planned regular phone contacts every 2 weeks were performed by the patients. Acceptability was lower for the online video classroom sessions. Out of a total of 8 different contents, patients actively participated in an average of 4.7 (SD 5.8) sessions. Patients consulted the chronic disease self-management support modules available on their tablet an average of 11.2 (SD 15.2) times during the 3-month follow-up and spent an average total time of approximately 16 hours on these consultations (median 5 hours).

**Table 4:** Acceptability of the nomhad eHealth system by the patients.

Variable		All patients (n=23)
**Follow-up**		
	Duration of follow-up period (days), mean (SD)	85.8 (19.0)
	Duration of effective follow-up period (days), mean (SD)	78.7 (24.2)
	Duration of system temporary suspension (days), mean (SD)	7.2 (14.4)
	Ratio of duration of effective follow-up to duration of follow-up period (%), mean (SD)	90.1 (18.2)
**Planned regular phone contacts with the** **call center nurse**		
	Number of actual planned phone contacts, mean (SD)	3.5 (1.4)
	Number of planned phone contacts scheduled, mean (SD)	3.7 (0.8)
	Ratio of actual to planned number of phone contacts (%), mean (SD)	87.3 (34.6)
**Online video classroom sessions**		
	Number of attended sessions (any type), mean (SD)	4.7 (5.8)
	Total duration of actual participation (minutes), mean (SD)	136.3 (206.7)
	Ratio of duration of actual participation to overall session duration (%), mean (SD)	60.5 (39.6)
**Information modules**		
	Number of consultations, mean (SD)	11.2 (15.2)
	Total duration of consultations (minutes), median (Q1-Q3)	310.4 (3-1561)

The program also appeared to be feasible. No eligible patients refused to participate in the study. One patient withdrew consent after 1 week of follow-up but no patients discontinued prematurely due to lack of connection or inability to use the study equipment. Eight patients (35%) required at least one maintenance visit and 6 patients (26%) needed at least one device replacement or had technical issues with the study equipment. Data transmission failures were mostly due to connection issues related to the mountainous study area, and represented an average of 3.4% (SD 5.9%) of the total number of data transmissions per patient.

### Safety Evaluations

Nine patients (39%) experienced a total of 16 AEs during their follow-up period. None of these AEs were considered to be related to the study medical device or procedures and no study discontinuation resulted from any AE. Seven patients (30%) experienced a total of nine serious AEs (Table 5), all of which were related to unscheduled hospitalizations. All these serious AEs were considered by the investigators to be related to the chronic diseases under investigation or to other diseases. The most frequent serious AEs were cardiac failures (3/23, 13%).

**Table 5:** Serious adverse events occurring during study follow-up.

Sex/age	Chronic diseases	Serious adverse event (MedDRA-preferred term)^a^	Time from inclusion to onset (days)	Duration (days)	Severity	Time since last ORI^b^ (days)	Last color of ORI^c,d^	Last color of SSI^e,f^
Male/82 years	CHF^g^/diabetes	Cardiac failure	32	4	Moderate	2	Yellow	SSIH^h^: Blue SSIR^i^: Green SSIP^j^: Green SSID^k^: Green SSIT^l^: Green SSIM^m^: Green
Male/62 years	CHF/diabetes	Cardiac failure/erysipelas	52	20	Severe	24^n^	Yellow	SSIH: Green SSIR: Green SSIP: —^o^ SSID: Green SSIT: — SSIM: Green
Male/72 years	CHF/diabetes	Atrial fibrillation	38	8	Moderate	2	Yellow	SSIH: Green SSIR: Blue SSIP: Green SSID: Orange SSIT: Green SSIM: Green
Male/84 years	CHF/COPD/diabetes	Cardiac failure	38	14	Severe	1	Yellow	SSIH: Blue SSIR: Green SSIP: Green SSID: Orange SSIT: Green SSIM: Green
Female/66 years	CHF/COPD/diabetes	COPD/alveolitis	86	11	Moderate	9	Yellow	SSIH: Green SSIR: Green SSIP: Green SSID: Green SSIT: — SSIM: Green
Male/62 years	CHF/COPD/diabetes	Urethral stenosis	31	5	Moderate	1	Yellow	SSIH: Blue SSIR: Green SSIP: — SSID: Green SSIT: — SSIM: Green
Male/64 years	CHF/COPD/diabetes	Arterial rupture	42	1	Moderate	5^p^	Orange	SSIH: Green SSIR: Orange SSIP: Orange SSID: Yellow SSIT: Green SSIM: Green

^a^Serious adverse events were coded using the MedDRA dictionary version 19.1.

^b^ORI: overall risk indicator.

^c^Last color of ORI is a weighted combination of the SSI status with specific calculation rules in case of missing data, depending on the time interval since their last available transmission.

^d^Green ORI, no action required; yellow ORI, low-priority action; orange ORI, medium-priority action; and red ORI, high-priority action.

^e^SSI: stability system indicator.

^f^Green SSIs indicate stability, whereas blue, yellow, orange, and red SSIs indicate decreasing stability, from slightly to markedly unstable. Blue SSI triggers increased frequency of data measurements.

^g^CHF: chronic heart failure.

^h^SSIH: hemodynamic stability system indicator.

^i^SSIR: respiratory stability system indicator.

^j^SSIP: pain/sleep quality stability system indicator.

^k^SSID: diet/diuresis stability system indicator.

^l^SSIT: tissue integrity stability system indicator.

^m^SSIM: medication stability system indicator.

^n^Patient was in the NOMHAD suspension period at his own request.

^o^Not available.

^p^Patient was in the NOMHAD suspension period due to programmed hospital admission.

## Discussion

### Principal Findings

This exploratory study demonstrated that the NOMHAD eHealth system was accurate in generating ORIs that reflect health status changes in patients with multimorbidity. The ORIs generated by the system agreed well with the ORIs that were expected when they were recalculated using the raw data teletransmitted. Sensitivity and specificity were both high for the ORIs and for the individual clinical stability system indicators. These results indicate that the system provided an efficient and accurate means of transmitting health status data and that the ORIs generated by the algorithm accurately reflected the health stability of the patients.

All study patients generated telenotifications, most of which were of low or moderate priority. Less than one-third of the patients required a high-priority telecoaching action. However, because ORIs were systematically recalculated every time new data were teletransmitted, a high number of ORIs (N=6263) were generated. This total was almost 3 times higher than what was anticipated for 23 patients over the study duration (100 days), assuming 1 telenotification per patient per day (~2300 ORIs). This disparity reflected the conservative nature in which the telenotifications were programmed to assure the safety of these patients with multimorbidity, which was a main priority during this study. In addition, each time a patient variable was out of the expected normal range, the self-measurement had to be repeated. Another reason for the high number is that some patients became high consumers of the system and performed their own repeated measurements every day. This could also explain why 71.53% (4480/6263) of the ORIs were of low (yellow) priority. A high number of ORIs were generated, leading to a substantial nurse workload, as a median of 21 outgoing telenotification management–related contacts were made to each patient over the 3-month follow-up. In managing the telenotifications, the call center nurses were able to effectively filter those requiring high-priority telecoaching actions. It is anticipated that future technical developments will reduce the number of low-priority ORIs.

The study showed that the NOMHAD eHealth system was generally well accepted by patients, nurses, and physicians, all of whom indicated high levels of satisfaction with most aspects of the program and a willingness to use the system long term. Responses to the self-questionnaires indicated that the system achieved a key aim of telehealth programs: to better manage and coordinate timely care for patients with multiple chronic diseases. Importantly, most users felt that it helped patients to better manage their diseases and symptoms and better understand their illnesses. Nurses felt that it helped them to communicate with patients more effectively, whereas most physicians felt that it helped them to better manage their patients. Most of the physicians also felt that the patients seen in consultation were only those who truly needed physician intervention. For patients, regular contact with the call center nurses was considered an important aspect of the program, and many of them considered this as the best part. Chronic diseases often limit social contact, especially for patients who live alone. It appears that contact with the nurses provided a key social interaction for many patients.

Patient satisfaction and participation were lower for the online video classroom sessions. Not all of the 8 different 40-minute sessions were relevant for every patient and attendance was relatively low. In addition, the mountainous terrain of the study setting caused some technical and connectivity issues, which affected the transmission quality of the videoconferences and may have frustrated some patients. By contrast, patients consulted the disease self-management support modules provided on their tablet approximately 11 times, indicating that they were interested in learning about their chronic diseases. These support modules were likely considered more convenient because patients could consult these at any time, unlike the online video classroom sessions, which were scheduled weekly at fixed times.

A recent study also investigated the views and experiences of patients and health care personnel using a telehealth and online self-management program for patients with multiple chronic diseases (diabetes, cardiovascular disease, and hypertension) in the Renewing Health Project [33]. Although there were differences between the 2 programs, both focused on patient-reported health status variables and on improving self-management skills. An important finding was that the expectations of patients, who expected feedback and support, differed from those of the health care staff, who expected the program to make patients more independent and able to perform self‑management tasks without staff support. By contrast, the regular every 2-week patient–nurse contact, which provided consistent feedback and support, was a key aspect of the NOMHAD program and highly regarded by the patients. Together, these findings suggest that telehealth programs should provide a means of maintaining a level of personal contact that preserves patient engagement. Insufficient contact may result in patients feeling isolated or abandoned.

### Strengths

This study has several strengths. Whereas most previous telehealth programs have focused on a single disease, the NOMHAD eHealth system was designed for patients with multiple chronic diseases. With multimorbidity on the rise, the study patients likely represent a more realistic “real-world” situation than those studied in many other telehealth programs. In addition, patients were living in a rural mountainous area, whereas the call center nurses were nearly 750 km away and the physicians were in private practice or a hospital nearby. Despite some connectivity issues, which are to be expected in such settings, this study demonstrated the system’s feasibility in remote conditions where many patients may face medical isolation. The telenotification accuracy of the system was verified, both for overall assessments of health status and for the individual clinical stability systems indicators. In addition, the NOMHAD eHealth system collects a comprehensive array of patient data that include subjective assessments of well-being, as well as objective self-measurements of patient health variables.

### Limitations

Regarding limitations, the self-questionnaires were created specifically for this study and were not independently validated. Besides, very few nurses and physicians participated in the study. Therefore, the questionnaire results for these health care providers must be viewed with caution. Although the feasibility results were positive, the follow-up period was relatively short for a program designed to be used long term. These results need to be confirmed in a longer study. Other minor problems were that the standard glycemia thresholds were too narrow, which resulted in irrelevant notifications, and that the blood pressure cuffs were too small for some patients with obesity.

The NOMHAD eHealth system was derived from earlier Spanish versions of the NOMHAD Chronic software platform (version 1.5.2) and the NOMHAD Mobile app (version 1.2.0). The Spanish version was used in a 3-arm randomized clinical trial comparing a structured telephone intervention, with and without the NOMHAD eHealth system, with usual care in 472 elderly patients with multimorbidity [31]. In Spain, the telemonitoring was performed by a hospital nurse team, whereas in this study in France, it was based on call center nurses. After a 12-month follow-up, the main outcome of health-related quality of life was significantly higher with the telehealth intervention than with usual care (*P*<.001). No differences in mortality or health care utilization were found. Nevertheless, this earlier study also demonstrated that the NOMHAD eHealth system was feasible for long-term use [31].

The medical relevance of the medium- to high-priority telenotifications (orange and red ORIs) also needs to be evaluated because of the significant workload they generated for nurses. As the aim of the study was to support high-risk patients, those included in this study were highly comorbid, with a mean of 6.5 concomitant diseases, and had severe targeted diseases, documented by a low left ventricular ejection fraction, a low forced expiratory volume in 1 second, and high proportions of insulin-dependent patients or those requiring home oxygen or ventilator support. Moreover, 7 patients underwent an unscheduled acute hospital admission during their 3-month follow-up. This preliminary study was not designed to assess medical relevance of the generated ORIs or clinical benefits, which would require a much larger and longer randomized controlled trial. However, several recent studies of similar telehealth and telemonitoring programs in patients with multimorbidity have reported positive results, suggesting that such programs can reduce disease exacerbations and health care costs [34,35]. A program for patients with chronic conditions in Australia found that, compared with the previous year, the telemonitoring program reduced medical expenses by 46.3%, pharmaceutical expenses by 25.5%, unscheduled hospital admissions by 53.2%, hospital length of stay by 67.9%, and mortality by over 40% [34]. In Spain, the ValCrònic program for patients with multiple chronic conditions found significant reductions in body weight; significantly smaller proportions of patients with high systolic or diastolic blood pressure, or elevated glycosylated hemoglobin concentrations in patients with diabetes; and significant reductions in emergency service use and hospitalizations [35].

### Conclusions

In conclusion, this study met its principal objectives by providing evidence that the NOMHAD eHealth system is accurate, useful, acceptable, informative, and feasible to implement and use. These favorable outcomes depended on the commitment and coordination of all users and study staff, all of which were facilitated by the system network. Although the NOMHAD eHealth system is based on efficient teletransmission of electronic data and accurate algorithmic evaluation, the system also facilitates human interaction on a regular basis, which appeared to be one of its most important features. The system was also considered useful to support disease education and promote patient awareness toward a better understanding of health data. This study confirms that the system is feasible for larger, longer studies to investigate its impacts on clinical variables, patient-reported outcomes, and medical resources used. However, in their comprehensive meta-review, Elbert et al [36] concluded that, given the consistent effectivity of telehealth for most patients, greater attention should be given to intervention evaluation rather than to obtaining additional evidence for their efficacy.

## Abbreviations

AE: adverse event
CHF: chronic heart failure
COPD: chronic obstructive pulmonary disease
ORI: overall risk indicator
SSI: stability system indicator
